# Coronary artery disease severity and risk stratification of patients with non ST-elevation acute coronary syndrome using CHA_2_DS_2_-VASc-HSF score

**DOI:** 10.1186/s12872-024-03929-5

**Published:** 2024-05-21

**Authors:** Mohamed Aboel-Kassem F. Abdelmegid, Mariam Essa Fares Hanna, Salwa R. Demitry, Mohamed Aly Hassan Abdelhafez

**Affiliations:** 1 Cardiovascular Medicine Department, Assiut University Heart Hospital, Assiut University, Assiut, 71526 Egypt; 2 Department of Cardiology, Assiut Police Hospital, Assiut, 71514 Egypt

**Keywords:** CHA_2_DS_2_-VASc-HSF score, Syntax score, NSTE ACS, MACE, Coronary artery disease

## Abstract

**Background:**

Risk stratification assessment of patients with non-ST elevation acute coronary syndrome (NSTE ACS) plays an important role in optimal management and defines the patient’s prognosis. This study aimed to evaluate the ability of CHA_2_DS_2_-VASc-HSF score (comprising of the components of the CHA_2_DS_2_-VASc score with a male instead of female sex category, hyperlipidemia, smoking, and family history of coronary artery disease respectively) to predict the severity and complexity of CAD and its efficacy in stratification for major adverse cardiovascular events (MACE) in patients with NSTE ACS without known atrial fibrillation.

**Methods:**

This study included 200 patients (males 72.5%, mean age 55.8 ± 10.1 years) who were admitted with NSTE ACS. CHA_2_DS_2_-VASC-HSF score was calculated on admission. Patients were classified into three groups according to their CHA_2_DS_2_-VASC-HSF score: low score group (< 2; 29 patients), intermediate score group (2–4; 83 patients), and high score group (≥ 5; 88 patients). Coronary angiography was conducted and the Syntax score (SS) was calculated. Clinical follow-up at 6 months of admission for the development of MACE was recorded.

**Results:**

SS was significantly high in the high CHA_2_DS_2_-VASc-HSF score group compared with low and intermediate score groups. CHA_2_DS_2_-VASc-HSF score had a significant positive strong correlation with syntax score (*r* = 0.64, *P* < 0.001). Smoking, vascular disease, hyperlipidemia, and CHA_2_DS_2_-VASc-HSF score were independent predictors of high SS. For the prediction of severe and complex CAD, CHA_2_DS_2_-VASc-HSF score had a good predictive power at a cut-off value ≥ 5 with a sensitivity of 86% and specificity of 65%. Hypertension, vascular disease, high SS, and CHA_2_DS_2_-VASc-HSF score were independent predictors of MACE. CHA_2_DS_2_-VASC-HSF score ≥ 4 was identified as an effective cut-off point for the development of MACE with 94% sensitivity and 70% specificity.

**Conclusions:**

CHA_2_DS_2_-VASC-HSF score is proposed to be a simple bedside score that could be used for the prediction of the severity and complexity of CAD as well as a risk stratification tool for the development of MACE in NSTE ACS patients.

## Background

Despite therapeutic advances, coronary artery disease (CAD) remains the most common cause of death worldwide [1]. Patients with acute coronary syndrome (ACS) have a wide spectrum of risks for death and cardiovascular ischemic events [2]. Achieving optimal myocardial perfusion in patients with NSTEMI is important to minimize infarct size and improve long-term prognosis, as evidenced by studies on patients with STEMI [3, 4]. Early risk stratification assessment of patients with non-ST elevation acute coronary syndrome (NSTE ACS) plays an important role in their management and prognosis. The available risk stratification scores for NSTE ACS (TIMI, PURSUIT, and GRACE scores) are either complex or difficult to utilize in daily clinical practice [5–7]. Therefore, the risk stratification model should be simple, straightforward, and use clinical risk factors that already affect the disease.

**Table 1:** Definition of CHA_2_DS_2_-VASc-HSF score

Abbreviation	Definition	Points
C	Congestive heart failure	1
H	Hypertension	1
A_2_	Age ≥ 75 years	2
D	Type 2 Diabetes mellitus	1
S_2_	Stroke or transient ischemic attack	2
V	Vascular disease	1
A	Age 65–74 years	1
Sc	Sex category (male)	1
H	Hyperlipidemia	1
S	Smoking	1
F	Family history of coronary artery disease	1
Maximum score		12

CHA_2_DS_2_-VASc score proved to be effective for assessing the risk of stroke in non-valvular atrial fibrillation (AF) patients and guiding anti-thrombotic therapy [8]. CHA_2_DS_2_-VASc score components include similar risk factors for the development of CAD. Therefore, CHA_2_DS_2_-VASc score (with male instead of female in the sex category) is used as a predictor of the severity of CAD using the Gensini score in stable patients [9]. The CHA_2_DS_2_-VASc-HSF score (Table 1) was used to predict the severity of atherosclerosis using the SYNTAX score (SS) in patients with ST-elevation ACS [10]. However, there is insufficient data about using the CHA_2_DS_2_-VASc-HSF score for the prediction of the CAD severity and risk stratification of major adverse cardiovascular events (MACE) in NSTE ACS patients.

Therefore, this study aimed to evaluate the CHA_2_DS_2_-VASc-HSF score to predict the severity and complexity of CAD using SS and its effectiveness as a risk stratification tool for MACE in patients with NSTE ACS without known AF.

## Methods

### Study Population

This was a prospective observational study that was conducted at Assiut University Heart Hospital, Assiut, Egypt. It included all consecutive patients who were admitted to the Coronary Care Unit with NSTE ACS. Patients with a history of coronary artery bypass grafting surgery, significant valvular heart disease, AF, or ST-elevation myocardial infarction (STEMI) were excluded.

### Sample size calculation

Sample size calculation was carried out using G Power 3 software. A calculated minimum sample of 176 NSTE ACS patients was needed to detect an effect size of 0.1 in the HR (Hazard Ratio) for the severity of CAD/MACE, with an error probability of 0.05 and 80% power on a two-tailed test.

### Study design

On admission, all patients were subjected to a detailed medical history, physical examination, electrocardiography, and echocardiographic examinations with laboratory investigations. Then, the components of the CHA_2_DS_2_-VASc-HSF score were obtained for each patient. The CHA_2_DS_2_-VASc-HSF score was calculated by allocating one point for each of the following: the presence of chronic heart failure, hypertension, diabetes mellitus (DM), vascular disease, age 65–74 years, male gender as a sex category, hyperlipidemia, smoking, family history of CAD, and two points for the history of stroke or transient ischemic attack and age ≥ 75 years (Table 1). Patients were classified according to CHA_2_DS_2_-VASc-HSF score into three groups: Low (< 2), intermediate (2–4), and High (≥ 5) score.

A coronary angiogram was performed for all patients either during the hospital stay for high-risk patients or within three months after discharge. The complexity of CAD was assessed using SS [11]. It was calculated for each patient by scoring all the coronary artery lesions with diameter stenosis ≥ 50% in vessels ≥ 1.5 mm using a web-based (http://www.syntaxscore.org) or smartphone application. Patients were divided into three tertiles according to the SS: low (≤ 22), intermediate (23 to 32), and high SS tertile (≥ 33).

All patients were followed up for 6 months after discharge using outpatient visits and/or telephone contacts. During follow-up, MACE was recorded.

### Definitions

NSTE ACS was defined as presenting with acute typical chest pain with at least one of the following characteristics: (1) recent significant ECG changes that include transient ST-segment elevation, persistent or transient ST-segment depression, T-wave inversion, flat T waves or pseudo-normalization of T waves, (2) positive cardiac enzymes, and (3) prior existence of CAD [12].

Hyperlipidemia was considered to be low-density lipoprotein cholesterol above the target level according to the National Cholesterol Education Program-III recommendations [13] or the use of anti-hyperlipidemic medications. Smoking was defined as daily smoking of any number of cigarettes for at least 1 year. Family history of CAD was defined as the presence of CAD in any first-degree relative: mother, father, siblings, or child, irrespective of age [14].

MACE was defined as the composite of death, nonfatal myocardial infarction (MI), nonfatal stroke, or hospitalization for unstable angina.

### Statistical analysis

Continuous variables were expressed as mean ± standard deviation (SD) and categorical variables as number and frequency (%). Chi-Square test was used to compare the three groups for categorical variables. For continuous variables, Shapiro-Wilk test was used to test the normality of data. Then, one-way ANOVA test for normally distributed data and Kruskal–Wallis test for non-normally distributed data were used to compare the mean/median between the three groups. Correlation was performed using Spearman Rank correlation coefficient. Receiver operator characteristics (ROC) curve was analyzed as the area under the curve (AUC), and 95% confidence interval (CI) and was used to determine the sensitivity and specificity of the CHA2DS2-VASc-HSF score and its cut-off value for predicting the severity and complexity of CAD and MACE. The cumulative event-free survival curves for MACE were constructed using the Kaplan-Meier method and were compared by the Log-rank test. Multicollinearity was assessed before multivariable regression model building using simple correlation (*r* > 0.2), variance inflation factor (VIF) (> 3), and likelihood backward regression technique to exclude variables with high correlation. Significant variables in univariate logistic regression were then adjusted in multivariate analysis to obtain significant independent predictors of the severity of CAD and to calculate the Odds Ratio (OR) and adjusted OR with a 95% CI. The clinical and demographic factors with proven statistical significance from the univariate analyses were further included in multivariable Cox hazard regression analysis to identify the independent predictors of MACE including Hazard Ratio (HR), adjusted HR, and 95% CI. A *p*-value of < 0.05 was considered statistically significant. All statistical analyses were performed using IBM-SPSS Statistics for Windows, version 24.0 (IBM Corp., Armonk, NY, USA).

## Results

**Table 2:** Patients’ characteristics and MACE of the studied population

Variable	Total number (200 patients)
Age (Years)	55.84 ± 10.13
Congestive heart failure	24 (12)
Hypertension	118 (59)
Age ≥ 75 years	7 (2.5)
DM	97 (48.5)
Prior stroke or TIA	5 (2.5)
Vascular disease	80 (40)
Age (65–74 years)	42 (21)
Male sex	145 (72.5)
Hyperlipidemia	147 (73.5)
Smoking	117 (58.5)
Family history of CAD	54 (27)
Clinical Presentation	
Unstable Angina	155 (77.5)
NSTEMI	45 (22.5)
MACE	16 (8)

Data are expressed in the form of mean ± SD or frequency (%).

CAD: coronary artery disease; MACE: major adverse cardiovascular events; NSTEMI: non-ST elevation myocardial infarction; DM: diabetes mellitus; TIA: transient ischemic attack.

A total of 200 patients with NSTE ACS without AF (mean age: 55.84 ± 10.13 years) were included in this study. Table 2 shows that the majority of the studied patients were males (72.5%) and had hyperlipidemia (73.5%). Moreover, the most frequent domains of CHA_2_DS_2_-VASc-HSF were hypertension (59%), smoking (58.5%), and DM (48.5%). The majority of patients, 155 patients (77.5%), presented with unstable angina ACS and negative Troponin test. The majority of the patients were treated conservatively (179 patients, 89.5%).

**Table 3:** Patients’ characteristics and MACE among the three groups according to CHA_2_DS_2_-VASc-HSF score

Variables	Low score group (29 patients)	Intermediate score group (83 patients)	High score group (88 patients)	*p*-value
Age (years)	51.93 ± 11.28	54.10 ± 8.91	58.76 ± 10.14	0.003
Congestive Heart Failure	0	5 (6.0)	19 (21.6)	0.001
Hypertension	11 (37.9)	44 (53.0)	63 (71.6)	0.002
Age ≥ 75 years	0	0	7 (8.0)	0.01
DM	6 (20.7)	29 (34.9)	62 (70.5)	< 0.001
Prior stroke or TIA	0	1 (1.2)	4 (4.5)	0.24
Vascular disease	0	23 (27.7)	57 (64.8)	< 0.001
Age (65–74 years)	4 (13.8)	13 (15.7)	25 (28.4)	0.07
Male sex	10 (34.5)	60 (72.3)	75 (85.2)	< 0.001
Hyperlipidemia	4 (13.8)	56 (67.5)	87 (98.9)	< 0.001
Smoking	6 (20.7)	43 (51.8)	68 (77.3)	< 0.001
Family history of CAD	1 (3.4)	22 (26.5)	31 (35.2)	0.004
Syntax score	4.48 ± 6.57	12.71 ± 9.11	24.60 ± 11.80	< 0.001
MACE	0	3 (3.6)	13 (14.8)	0.006
Death	0	0	1 (1.1)	0.528
Nonfatal MI	0	1 (1.2)	6 (6.8)	0.074
Nonfatal stroke	0	1 (1.2)	4 (4.5)	0.243
Hospitalization for UA	0	1 (1.2)	2 (2.3)	0.655

Data are expressed in the form of mean ± SD or frequency (%).

CAD: coronary artery disease; DM: diabetes mellitus; MACE: major adverse cardiovascular events; MI: myocardial infarction; TIA: transient ischemic attack; UA: unstable angina.

**Fig. 1:**
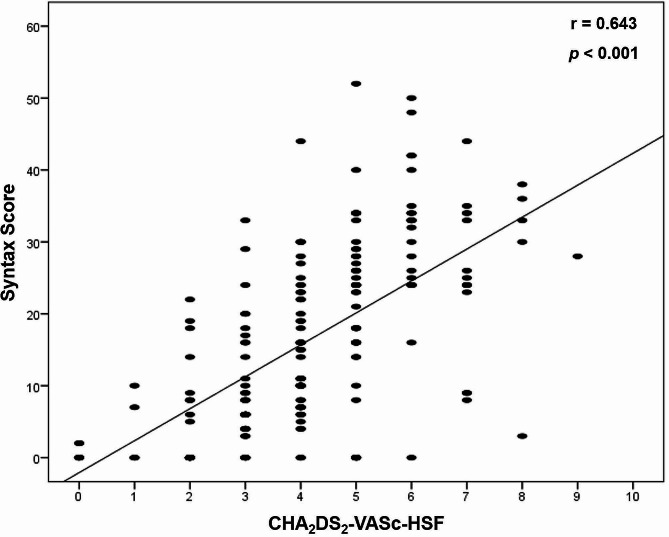
Correlation between CHA_2_DS_2_-VASc-HSF score and Syntax Score

Table 3 demonstrates that the frequency of hypertension, DM, and a history of congestive heart failure significantly increased as the CHA_2_DS_2_-VASc-HSF score increased. Besides, the prevalence of hyperlipidemia and vascular disease was significantly higher in the high score group than in lower and intermediate score groups (*p* < 0.001 for each). Moreover, there was a significant difference among the CHA_2_DS_2_-VASc-HSF score groups regarding SS and MACE (*p* < 0.001 and 0.006, respectively). Furthermore, it was noticed that the CHA_2_DS_2_-VASc-HSF score had a significant positive correlation with the syntax score (*r* = 0.643; *P* < 0.001) (Fig. 1).

**Table 4:** Patients’ characteristics among the three tertiles according to Syntax score

Variables	Low tertile group (122 patients)	Intermediate tertile group (43 patients)	High tertile group (35 patients)	*p*-value
Age (years)	54.07 ± 10.17	57.93 ± 9.50	59.40 ± 9.58	0.007
Congestive Heart Failure	7 (5.7)	11 (25.6)	6 (17.1)	0.002
Hypertension	69 (56.6)	27 (62.8)	22 (62.9)	0.68
Age ≥ 75 years	2 (1.6)	2 (4.7)	3 (8.6)	0.13
DM	46 (37.7%)	28 (65.1%)	23 (65.7)	0.001
Prior stroke or TIA	2 (1.6)	2 (4.7)	1 (2.9)	0.55
Vascular disease	31 (25.4)	25 (58.1)	24 (68.6)	< 0.001
Age (65–74 years)	21 (17.2)	11 (25.6)	10 (28.6)	0.25
Male sex	82 (67.2%)	34 (79.1%)	29 (82.9%)	0.1
Hyperlipidemia	72 (59%)	43 (100%)	32 (91.4%)	< 0.001
Smoking	58 (47.5%)	31 (72.1%)	28 (80%)	< 0.001
Family history of CAD	29 (23.8%)	11 (25.6%)	14 (40%)	0.16
CHA_2_DS_2_-VASc-HSF	3.47 ± 1.50	5.33 ± 1.25	5.60 ± 1.45	< 0.001

Data are expressed in the form of mean ± SD or frequency (%).

CAD: coronary artery disease; DM: diabetes mellitus; TIA: transient ischemic attack.

**Table 5:** Univariate and multivariate logistic regression analysis of predictors for high Syntax score

Variables	Univariate analysis			Multivariable analysis		VIF
	OR (95% CI)	*p*-value	Adjusted OR (95% CI)		*p*-value	
Age (years)	1.045 (1.006–1.085)	0.024				0.7
Smoking	3.416 (1.413–8.260)	0.006	2.264 (1.021–6.118)		0.036	1.2
DM	2.357 (1.100–5.052)	0.028				2.1
Vascular disease	4.274 (1.941–9.292)	< 0.001	2.254 (1.117–4.321)		0.038	1.4
Hyperlipidemia	4.638 (1.357–9.835)	0.014	1.654 (1.009–3.019)		0.044	0.9
Family history of CAD	2.083 (0.970–4.474)	0.060				0.5
High CHA_2_DS_2_-VASc-HSF score	11.069 (4.075–20.066)	< 0.001	8.584 (2.691–17.285)		< 0.001	1.5

CAD: coronary artery disease; CI: confidence interval; DM: diabetes mellitus; OR: Odds ratio; VIF: variance inflation factor

**Table 6:** Univariate and multivariate Cox Hazard regression analysis of predictors for MACE

Variables	Univariate analysis		Multivariable analysis		VIF
	HR (95% CI)	*p*-value	Adjusted HR (95% CI)	*p*-value	
Hypertension	5.115 (1.162–12.506)	0.031	4.912 (1.115–8.457)	0.034	1.1
DM	2.416 (0.839–6.965)	0.10			1.4
Vascular disease	3.528 (1.226–8.155)	0.019	4.052 (1.393–11.787)	0.01	1.2
Hyperlipidemia	3.382 (0.321–7.166)	0.10			2.3
High SYNTAX score	1.760 (1.003–3.086)	0.049	3.201 (1.097–5.598)	0.038	0.8
High CHA_2_DS_2_-VASc-HSF score	5.125 (1.588–11.547)	0.006	4.324 (1.327–8.082)	0.015	1.8

CI: confidence interval; DM: diabetes mellitus; HR: Hazard ratio; VIF: variance inflation factor

**Fig. 2:**
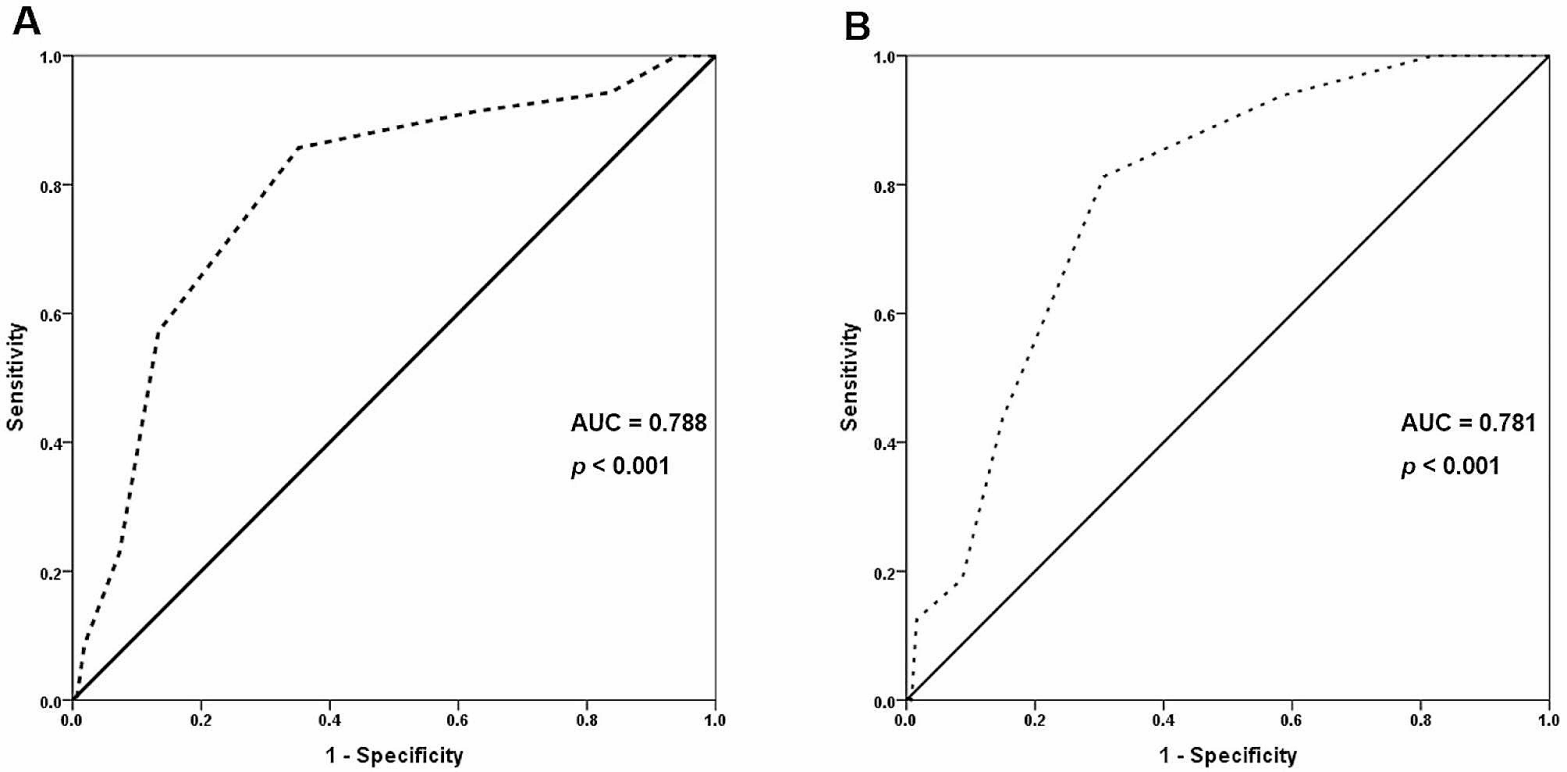
ROC curve for prediction of high Syntax score (**A**) and MACE (**B**) based on CHA_2_DS_2_-VASc-HSF score

**Fig. 3:**
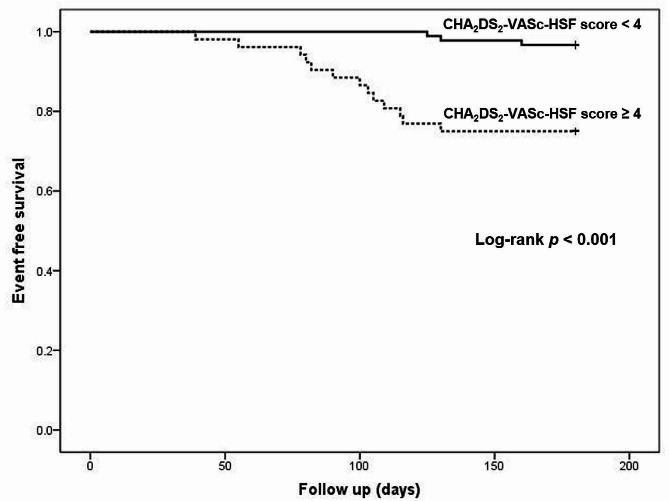
Kaplan-Meier survival curve for MACE as stratified for CHA_2_DS_2_-VASc-HSF score < 4 and ≥ 4

Table 4 illustrates that the mean age of patients in the high SS tertile was significantly higher than that in the intermediate and low SS tertiles (p 0.03 and 0.006, respectively). Moreover, the prevalence of a history of congestive heart failure and vascular disease was higher in the high SS tertile than in the other tertiles. Also, CHA_2_DS_2_-VASc-HSF score was significantly elevated in the high and intermediate SS tertiles compared with low SS tertiles (*p* < 0.001 for each). However, there was no significant difference in CHA_2_DS_2_-VASc-HSF score between high and intermediate SS tertiles (*p* = 0.40). Multivariate logistic regression analysis revealed that smoking, vascular disease, hyperlipidemia, and CHA_2_DS_2_-VASc-HSF score were independent predictors of high SS (*p* = 0.03, 0.03, 0.4, and < 0.001, respectively) (Table 5). The cut-off value of the CHA_2_DS_2_-VASc-HSF score for predicting high SS was ≥ 5 with a sensitivity of 86% and specificity of 65% (AUC 0.788, 95% CI 0.704–0.872, *p* < 0.001) (Fig. 2A). Multivariate Cox regression analysis revealed that hypertension, vascular disease, and high SS, as well as CHA_2_DS_2_-VASc-HSF score, were predictors of MACE in the current study (Table 6). At a cut-off point of ≥ 4, the CHA_2_DS_2_-VASc-HSF score had 94% sensitivity and 70% specificity for the prediction of the development of MACE (AUC 0.781, 95% CI 0.680–0.882, *P* < 0.001) (Fig. 2B). Therefore, the patients were classified according to CHA_2_DS_2_-VASc-HSF score cut-off point of ≥ 4 and Kaplan-Meier survival analysis showed that MACE free survival rate was higher in CHA_2_DS_2_-VASc-HSF score ≥ 4 group than < 4 (*P* < 0.001) (Fig. 3).

## Discussion

In patients with NSTE ACS without known AF, the present study reported that CHA_2_DS_2_-VASc-HSF score was significantly higher in severe and complex CAD than mild and moderate CAD. Moreover, the CHA_2_DS_2_-VASc-HSF score had a significant positive strong correlation with the severity of CAD. Besides, the CHA_2_DS_2_-VASc-HSF score was an independent predictor of severe and complex CAD with a cut-off value of CHA_2_DS_2_-VASc-HSF score ≥ 5 for predicting severe CAD.

Age, gender, hypertension, smoking, hyperlipidemia, and diabetes mellitus are the well-known major risk factors for CAD [1]. The majority of CAD patients have more than one risk factor and the more combination of these risk factors the high risk for CAD [15, 16]. Therefore, the CHA_2_DS_2_-VASc-HSF score’s components promote atherosclerosis and are linked to CAD severity.

Cetin et al. evaluated the validity of CHADS_2_, CHA_2_DS_2_-VASc scores, and the newly defined CHA_2_DS_2_-VASc -HS score by adding hyperlipidemia, smoking, and male instead of the female gender to identify those who at high risk of severe CAD [9]. They found that the CHADS_2_, CHA_2_DS_2_-VASc, and particularly CHA_2_DS_2_-VASc -HS scores were positively correlated with CAD severity. Moreover, they found that CHA_2_DS_2_-VASc-HS score was the best scoring system to predict CAD severity in stable CAD patients. Other studies reported the same findings in stable CAD which enrolled a large number of studied patients and included the family history of CAD to the score to be CHA_2_DS_2_-VASc -HSF [17, 18]. However, our study focused on NSTE ACS patients who are a high-risk group of patients. Besides, the current study added the family history of CAD to the score which is shared by 27% as a risk factor that increases the likelihood prediction of CAD severity. The previous studies [9, 17, 18] used the number of diseased coronary arteries and the Gensini score to assess the CAD severity which depends on the presence of > 50% stenosis in a coronary artery which may not be indicative of the clinical importance of the CAD severity. This differs from our study which used the SS which assesses the lesion characteristics including complexity such as bifurcation and osteal locations, morphology, and location on the coronary artery reflecting the severity and complexity of the CAD. However, Gensini score does not put these lesion characteristics into consideration.

In patients with STEMI who underwent primary percutaneous coronary intervention, Uysal and his coworkers assessed the predictive value of CHA_2_DS_2_-VASc-HSF score for CAD severity as assessed by SS in patients with ST-elevation ACS [10]. They observed that the CHA_2_DS_2_-VASc-HSF score was an independent predictor of high SS ≥ 21 (OR 1.258, 95% CI 1.026–1.544, *p* = 0.028) together with age and ejection fraction. They concluded that the CHA_2_DS_2_-VASc-HSF score can predict atherosclerosis severity in patients with STEMI and also proved that factors comprising the CHA_2_DS_2_-VASc-HSF score promote atherosclerosis and were associated with the severity of CAD. Similar results were reported by another study conducted on ST-elevation ACS patients that showed CHA_2_DS_2_-VASc-HSF score were positively correlated with high SS and the cut-off value CHA_2_DS_2_-VASc-HSF score was ≥ 4 with 84.4% sensitivity and 81.9% specificity (AUC 0.83, 95% CI 0.746–0.915, *p* < 0.001) [19]. Another study demonstrated that CHA_2_DS_2_-VASc-HSF score was an independent risk factor for high Gensini score and multivessel CAD in patients with ACS including unstable angina, non-ST-elevation myocardial infarction (NSTEMI), or STEMI [20]. These results are in agreement with the results of our study but the main difference was that our study was conducted on NSTE-ACS patients and using SS to assess the severity of CAD.

Taşolar et al. assessed the accuracy of the CHA_2_DS_2_-VASc-HS score in identifying the severity and complexity of CAD using SS in NSTE-ACS patients [21]. They found that the CHA_2_DS_2_-VASc-HS score was high in high SS tertile compared with low and intermediate tertile. Furthermore, ROC curve analysis based on a high SS > 32, the cut-off value of the CHA_2_DS_2_-VASc-HS score was ≥ 5 with a sensitivity of 61.9% and specificity of 79.5% (AUC 0.781, 95% CI 0.725–0.831, *p* < 0.001). These results were similar to our results; however, patients with AF and previous percutaneous coronary intervention were enrolled as SS had been validated for native coronary arteries. Moreover, the score was formulated without adding the family history of CAD as a risk factor.

Clinicians need simple, objective, and quantitative tools to identify the patient at high risk and recommend the best prevention strategy. The current study showed that hypertension, vascular disease, and high SS, as well as CHA_2_DS_2_-VASc-HSF score, were predictors of MACE at 6 months. The cut-off value of the CHA_2_DS_2_-VASc-HSF score for predicting the development of MACE was ≥ 4. Each of the components of the CHA_2_DS_2_-VASc-HSF score has been reported as an independent predictor of adverse outcomes in patients with CAD [22–24]. Therefore, it was not surprising that there was an association between the CHA_2_DS_2_-VASc-HSF score and MACE in our study.

The association of CHADS_2_ and CHA_2_DS_2_-VASc scores with adverse events in patients with ACS was investigated in a large observational prospective multicenter study which was conducted at 39 hospitals in Taiwan [25]. Totally 3183 patients with ACS including STEMI, unstable angina, and NSTEMI were enrolled. The primary endpoint was the occurrence of adverse events which included subsequent myocardial infarction, stroke, or death at 1-year follow-up. They reported that CHA_2_DS_2_-VASC score of > 2 was associated with a higher rate of adverse events than those with a score of ≥ 2. Moreover, the CHA_2_DS_2_-VASc score had better diagnostic performance in predicting the composite endpoint as compared with the CHADS_2_ score. In contrast to our study, the previous study enrolled all ACS patients including STEMI patients, and used CHADS_2_ and CHA_2_DS_2_-VASC scores. However, the current study focused on NSTE ACS patients and used the CHA_2_DS_2_-VASC-HSF score by adding hyperlipidemia, smoking, and the family history of CAD which increases the predictive value of the score in assessing the risk of subsequent adverse events. Nevertheless, this multicenter study showed similar results to the present study.

A sub-analysis of SHINANO registry assessed the clinical validation of the CHA_2_DS_2_-VASc score for prognostic risk stratification in patients with CAD including stable, unstable angina, STEMI, and NSTEMI [26]. The primary endpoint was MACE which included death, non-fatal MI, and ischemic stroke at 1 year. It showed that the incidence of MACE was significantly higher in patients with CHA_2_DS_2_-VASc score ≥ 5 and the CHA_2_DS_2_-VASc score was an independent predictor for MACE (HR 1.26, 95% CI 1.15–1.39, *p* < 0.001) as we found in the current study. Another retrospective study included all patients who underwent percutaneous coronary intervention in a tertiary medical center over 10 years and the relation between the CHA_2_DS_2_-VASc score and clinical outcomes (the primary outcome was all-cause mortality and the secondary outcome was mortality or nonfatal MI) at 1 and 5 years were assessed [27]. It was reported that the primary and secondary outcomes at 1 and 5 years were significantly more frequent as the CHA_2_DS_2_-VASc score increased. Moreover, CHA_2_DS_2_-VASc score predicted the primary and secondary outcomes in a significant (*p* < 0.001) and linear fashion. However, our study assessed the CHA_2_DS_2_-VASc-HSF score and enrolled NSTE ACS patients with follow-up at 6 months but 54.4% of cases in the SHINANO registry [26] and 67.5% of patients in the other study [27] were due to ACS including STEMI with a long duration of follow up at 1 year and 5 years, respectively. Even so, these results were in agreement with our results.

Other studies evaluated the effectiveness of the CHA_2_DS_2_VASc score as a long-term predictor for prognosis in STEMI patients irrespective of the presence of AF [28, 29]. Bozbay and coworkers found that in-hospital cardiovascular mortality and long-term mortality were significantly frequent in the high CHA_2_DS_2_-VASc score group and admission CHA_2_DS_2_-VASc score > 2 was cut-off point for long-term mortality (AUC 0.821, 95% CF 0.76–0.89, *P* < 0.001) [28]. KORMI registry concluded that as the CHA_2_DS_2_VASc risk score increased, the incidence of adverse cardiac events was higher at 1, 6, 12, and 24 months and the CHA_2_DS_2_VASc risk score was an independent predictor for the long-term prognosis (*p* < 0.001) [29]. These results were not far away from our results in the current study but on STEMI patients and our study on NSTE-ACS.

There were a few limitations in the present study. This study was a single-center study with a small number of patients and a short period of follow-up. Therefore, a larger population is required with a further longer follow-up study is required to evaluate the long-term impact of the CHA_2_DS_2_-VASc HSF score on MACE. Besides, the treatment strategy of NSTE ACS (conservative medical treatment or invasive revascularization) does not take into consideration. However, the majority of the studied patients were treated conservatively.

## Conclusions

The CHA_2_DS_2_-VASc-HSF score is strongly correlated with and able to predict the severity and complexity of CAD in NSTE ACS patients without known AF. Furthermore, this score can be used as an effective risk stratification tool to predict short-term clinical outcomes in those patients. As the CHA_2_DS_2_-VASc-HSF score is a simple, bedside, practical, easily remembered score and does not require complex software to calculate, it may play a valuable role as a predictive formula for NSTE ACS risk assessment in daily practice.

## Data Availability

The datasets used and/or analyzed during the current study are available from the corresponding author upon reasonable request.

## Abbreviations

ACS: Acute Coronary Syndrome
AF: Atrial Fibrillation
AUC: Area Under the Curve
CAD: Coronary Artery Disease
CI: Confidence Interval
DM: Diabetes Mellitus
HR: Hazard Ratio
MACE: Major Adverse Cardiovascular Events
MI: Myocardial Infarction
NSTE ACS: Non-ST Elevation Acute Coronary Syndrome
NSTEMI: Non-ST elevation Myocardial Infarction
OR: Odds Ratio
ROC: Receiver operator characteristics curve
SD: Standard Deviation
STEMI: ST-Elevation Myocardial Infarction
SS: Syntax Score
